# Genome-wide identification of alternative splicing associated with histone deacetylase inhibitor in cutaneous T-cell lymphomas

**DOI:** 10.3389/fgene.2022.937623

**Published:** 2022-09-06

**Authors:** Shirong Yu, Jingzhan Zhang, Yuan Ding, Xiaojing Kang, Xiongming Pu

**Affiliations:** ^1^ Xinjiang Medical University, Urumqi, China; ^2^ Department of Dermatology, People’s Hospital of Xinjiang Uygur Autonomous Region, Urumqi, China; ^3^ Xinjiang Clinical Research Center for Dermatologic Diseases, Urumqi, China; ^4^ Xinjiang Key Laboratory of Dermatology Research, Urumqi, China

**Keywords:** histone deacetylase inhibitors, cutaneous T-cell lymphomas, alternative splicing, RNA binding proteins, bioinformatics analysis

## Abstract

Cutaneous T-cell lymphomas (CTCLs) are a kind of non-Hodgkin lymphoma that originates from skin, which is difficult to treat with traditional drugs. Human histone deacetylase inhibitors (HDACi) targeted therapy has become a promising treatment strategy in recent years, but some patients can develop resistance to the drug, leading to treatment failure. There are no public reports on whether alternative splicing (AS) and RNA binding proteins (RBP) affect the efficacy of targeted therapy. Using data from the Gene Expression Omnibus (GEO) database, we established a co-change network of AS events and RBP in CTCLs for the first time, and analyzed the potential regulatory effects of RBP on HDACi-related AS events. The dataset GSE132053, which contained the RNA sequence data for 17 HDACi samples, was downloaded and clean reads were aligned to the human GRCh38 genome by hierarchical indexing for spliced alignment of the transcripts, allowing four mismatches. Gene expression levels were evaluated using exons per million fragments mapped for each gene. Student’s t-tests were performed to evaluate the significance of changes in ratios for AS events, and regulated alternative splicing events (RASEs) were defined as events with *p* values less than 0.05. To sort the differentially expressed genes functional categories, Gene Ontology terms and Kyoto Encyclopedia of Genes and Genomes pathways were identified using the KOBAS 2.0 server. The regulatory mechanisms of the RASEs and RBPs were evaluated using Pearson’s correlation coefficient. Seven indirect events of HDACi resistance or sensitivity were identified: NIR_5151_RP11-977G19.10, NIR_4557_IRAG2, NIR_11870_SUMO1, NIR_5347_ING4, NIR_17935_DNAJC2, NIR_17974_CBLL1, and NIR_422_SLC50A1. The potential regulatory relationships between RBPs and HDACi-sensitive RASEs were also analyzed. *LEPR* and *HNRNPAO* significantly affected NIR_11870_SUMO1, suggesting a potential regulatory relationship. Additionally, *CNN1* may regulate NIR_5347_ING4, *CNOT3* may regulate NIR_17935_DNAJC2, and *DQX1* and *LENG9* may regulate NIR_422_SLC5A1. Overall, our findings establish a theoretical foundation for the precise targeted treatment of CTCLs with HDACi.

## Introduction

Cutaneous T-cell lymphomas (CTCLs) are a group of non-Hodgkin lymphomas that originate in the skin and are characterized by monoclonal expansion of T lymphocytes. The World Health Organization and the European Organization for Cancer Research and Treatment divide CTCLs into various types according to their clinical and histological characteristics. Mycosis fungoides (MF) and Sezary syndrome (SS) are the two most common CTCLs, accounting for approximately 53% of all CTCLs (Siegel et al., 2000; Jawed et al., 2014). The typical early presentation of MF is erythema resembling eczema. As the disease progresses, the clinical manifestations gradually transit from the erythema stage to the plaque stage and finally enter the tumor stage; a malignant skin tumor develops and invades the circulatory, lymphatic, and visceral tissues (Lei et al., 2020; Weiner et al., 2021). In contrast, SS is considered a more aggressive form of the disease and is associated with shorter survival (Larocca and Kupper, 2019).

For patients with no systemic involvement in the early stage of disease, topical medication or physical therapy is mostly used, whereas in patients with advanced refractory disease, systemic therapy or combined therapy is mostly used. Novel breakthroughs in treatment have also been reported. Many studies have reported good curative effects of targeted therapies (Kempf and Mitteldorf, 2021). However, many patients develop resistance to therapeutic drugs, which leads to treatment failure. Cancer cells can utilize alternative splicing (AS) to drive tumorigenesis and evade the effects of anticancer drugs (Martinez-Montiel et al., 2018; Sciarrillo et al., 2020). However, AS can also alter the coding regions of drug targets, resulting in treatment resistance (Siegfried and Karni, 2018). In CTCLs, AS of multiple genes is related to the occurrence and development of disease, and regulation of AS plays an important role in this (Van Doorn et al., 2002; Koch et al., 2003; Bagdonaite et al., 2015; Zhu and Gao, 2017; Yamaguchi et al., 2019).

Compared with traditional chemotherapy, human histone deacetylase inhibitors (HDACi) have become a promising treatment strategy with good results in the treatment of CTCLs (Lopez et al., 2018). HDACi are epigenetic regulators that induce acetylation of proteins involved in the modulation of gene expression, cell growth, differentiation, and apoptosis; however, only a subset of patients with CTCLs (30–35%) are responsive to HDACi (Qu et al., 2017). Functional CHK1 and high levels of thioredoxin or pro-survival BCL-2 may contribute to cancer cell resistance against HDACi (Lee et al., 2012). Notably, some highly expressed markers, such as leukocyte-associated immunoglobulin-like receptor two protein, present in the plasma of patients with anti-HDACi CTCLs, can be used as potential predictors of HDACi resistance in CTCLs therapy (Andrews et al., 2019). Further studies are needed to improve therapeutic approaches for using HDACi, and the discovery of more molecular and predictive biomarkers of clinical response to HDACi is critical.

AS is a driver of post-transcriptional variation. Epigenetic changes and AS in specific genes may also render cancer cells resistant to chemotherapeutic drugs (Hnilicová et al., 2011). HDAC activity can influence splice-site selection (Lu and Chao, 2012). An understanding of the mechanisms by which AS leads to drug resistance may facilitate the development of novel approaches to combat resistance to cancer treatments. RNA-binding proteins (RBPs), being the essential binding partners of intracellular RNA, dynamically bind to RNA, forming various complexes, including ribonucleoprotein particles (Castello et al., 2013). Moreover, RBPs play important roles in the regulation of post-transcriptional gene expression and binding of precursor mRNAs to control AS, thereby regulating cell functions (Fu and Ares, 2014; Lorenzi et al., 2019; Qin et al., 2020). We speculated that differences in the AS patterns of some genes and their related RBPs in patients with CTCLs might be associated with resistance or sensitivity to HDACi. However, there are currently no public reports on whether AS and RBP affect the efficacy of HDACi targeted therapy in CTCLs. For this study, we downloaded the RNA-seq data of 17 HDACi samples from patients with HDACi-sensitive or resistant CTCLs from the GEO database, and identified and compared those using bioinformatics methods to address the aforementioned issue. For the first time, a co-change network of AS events and RBP was established in CTCLs, and the potential regulatory effects of RBP on HDACi-related AS events was clarified.

## Materials and methods

### Public data retrieval and processing

Public sequence data files (Gene Expression Omnibus number: GSE132053) on peripheral blood samples from patients with MF or SS who were sensitive or resistant to HDACi (romidepsin or vorinostat) were downloaded from the Sequence Read Archive (SRA). The NCBI SRA Tool fastq-dump was used to convert the SRA Run files to the fastq format. Raw reads were trimmed using FASTX-Toolkit to remove low-quality bases. The clean reads were then evaluated using FastQC (http://www.bioinformatics.babraham.ac.uk/projects/fastqc) (Wang et al., 2021).

### Read alignment and differentially expressed gene analysis

Clean reads were aligned to the human GRCh38 genome using hierarchical indexing for the spliced alignment of transcripts (Kim et al., 2013). Uniquely mapped reads were used to calculate read numbers and fragments per kilobase of exon per million mapped fragments (FPKM) for each gene. Then, FPKM was used to evaluate the expression levels of genes. DEseq2 software was used to analyze DEGs. The scale factor was used to explain the differences in library depth. DEseq2 was then used to estimate gene dispersion. Finally, the negative binomial distribution model was fitted to DEseq2, and the hypothesis was tested using Wald or likelihood ratio tests. The results determined whether a gene was differentially expressed based on the fold change (FC) and false discovery rate (FDR). The criteria for establishing significant differences in expression were as follows: FC ≤ 0.5 or ≥2, and FDR <0.05.

### AS analysis

AS events (ASEs) and regulated ASEs (RASEs) between samples were defined and quantified using the ABL pipeline as described previously (Jin et al., 2017; Xia et al., 2017). Ten types of ASEs were defined based on the splice junction reads: alternative three splice site (A3SS), alternative 5′ splice site (A5SS), exon skipping (ES), mutually exclusive five untranslated region (UTR), mutually exclusive exon, intron retention, cassette exon, mutually exclusive three UTR, A5SS + ES, and A3SS + ES ASEs (Yan et al., 2022). For sample pair comparison, Fisher’s exact test was used to determine statistical significance using the alternative reads and model reads of the samples as input data. The ratio of alternatively spliced reads to constitutively spliced reads between compared samples, known as the RASE ratio, was calculated. A RASE ratio greater than or equal to 0.15 and a *p* value less than or equal to 0.01 were set as the thresholds for RASE detection. Student’s t-test was performed to evaluate alterations in the ratios of AS events in the repetition comparison. Events with *p* values less than 0.05 were considered RASEs.

### Co-expression analysis

A catalog of 2142 human RBPs was retrieved and combined from four previous reports (Castello et al., 2012; Gerstberger et al., 2014; Castello et al., 2016; Hentze et al., 2018). To explore the regulatory mode between RASE and RBPs, Pearson’s correlation coefficients (PCCs) were calculated between the splicing ratio of RASE and expression level of RBPs; the correlations were classified as negative, noncorrelated, or positive based on the PCC value.

### Functional enrichment analysis

To sort out functional categories of DEGs, KOBAS 2.0 server was used to identify Gene Ontology (GO) terms and Kyoto Encyclopedia of Genes and Genomes (KEGG) pathways (Xie et al., 2011). The Benjamini–Hochberg procedure and hypergeometric tests were used to define the enrichment of each term.

### Other statistical analyses

The pheatmap package (https://cran.r-project.org/web/packages/pheatmap/index.html) was used to perform clustering based on Euclidean distances. Differences between two groups were compared using Student’s t-test.

## Results

### Data introduction and preprocessing

**FIGURE 1:**
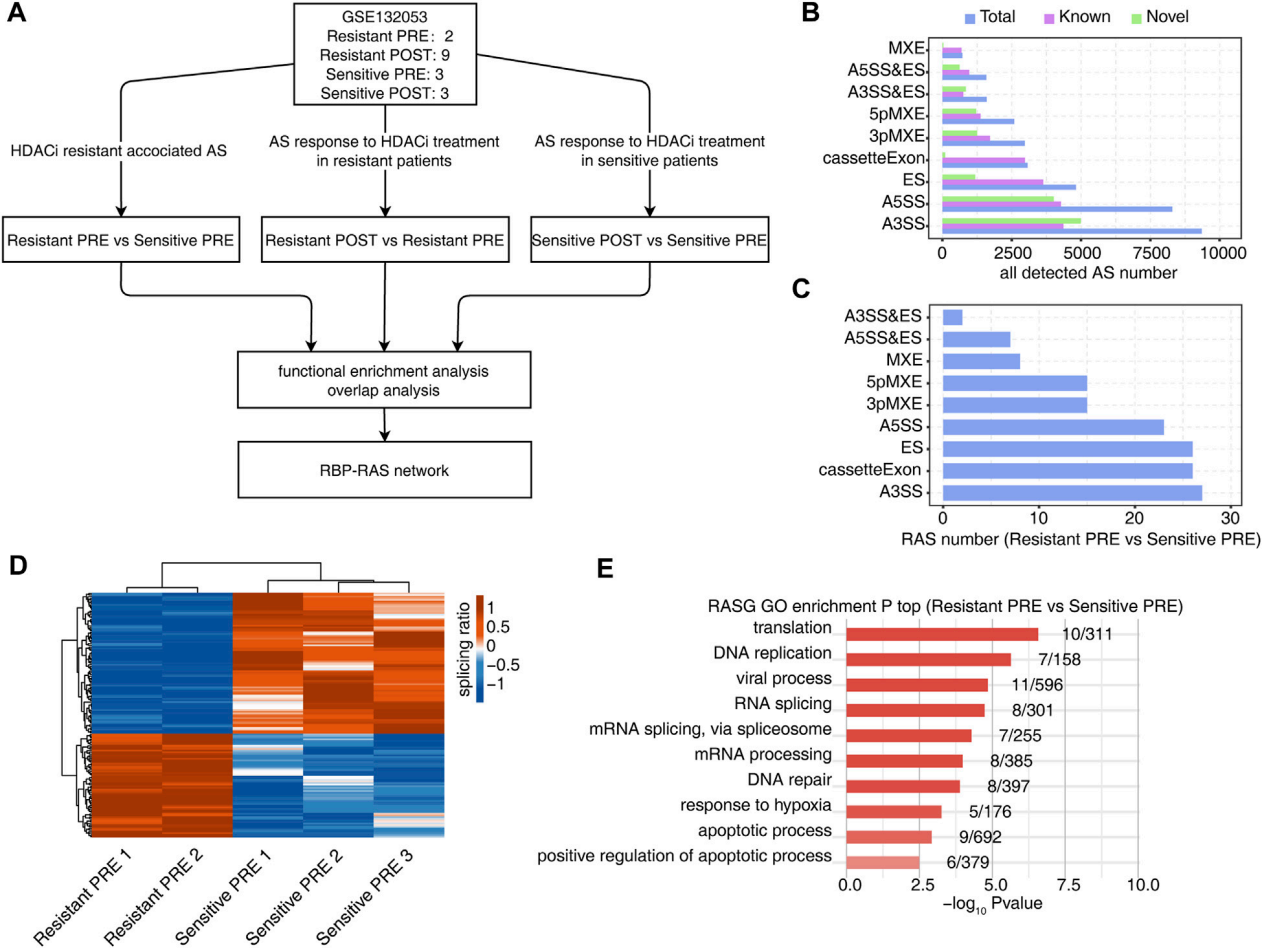
Identification of regulated AS events (RAS) between HDACi resistant and sensitive samples of peripheral blood from MF/SS patients before treatment with HDACi. **(A)** Workflow of bioinformatic analysis in this study. **(B)** The bar plot showing the number of all detected alternative splicing events (ASEs). *X*-axis: All ASE number. *Y*-axis: the different types of AS events. **(C)** The bar plot showing the number of all significant regulated alternative splicing events between Resistant PRE and Sensitive PRE samples. *X*-axis: NIR RASE number. *Y*-axis: the different types of AS events. **(D)** Hierarchical clustering heatmap of all significant RAS based on splicing ratio of Resistant PRE and Sensitive PRE samples. **(E)** Bar plot showing the most enriched GO biological process results of the NIR RAS between Resistant PRE and Sensitive PRE samples.

We downloaded the data on peripheral blood samples from patients with MF or SS who were sensitive or resistant to HDACi from the GEO database (GSE132053). Patients in the sensitive group experienced a partial or complete response, or disease stability after treatment with HDACi, whereas patients in the resistant group showed disease progression during HDACi treatment (Andrews et al., 2019). RNA-sequencing (RNA-seq) data for HDACi resistance included two cases before treatment and nine cases after treatment. RNA-seq data for HDACi sensitivity included three cases before treatment and three cases after treatment. RNA-seq data for 17 HDACi samples were identified and compared. The differential AS events (resistant AS, RAS) between the sensitive and resistant groups before treatment were analyzed and obtained. Variable splicing events with significant differences between the sensitive and resistant groups, and before and after treatment, were identified. The differential splicing events obtained in the previous two steps were further subjected to overlap analysis. A splicing regulatory network of RBPs/RASEs was constructed, and important genes were identified. The flowchart of the analysis is shown in Figure 1A.

### RASEs in HDACi resistant and sensitive groups before treatment with HDACi

All detected AS events are shown in Figure 1B, where ‘known’ represents an existing splicing event in the genome annotation file and ‘novel’ represents a newly identified splicing event. The analysis identified 149 differential AS events (resistant-RASEs) in the sensitive and resistant groups before treatment. Among these, ES/cassette exon, A5SS, and A3SS events were dominant (Figure 1C). The splicing ratio of differential splicing events was used to draw a heatmap, and there was a clear difference between the resistant and sensitive groups (Figure 1D). Functional pathway enrichment analysis of genes showing RASEs was performed using GO and KEGG pathways. These pathways were enriched in RNA splicing, DNA repair, hypoxia response, apoptosis, and other pathways closely related to HDACi-induced acetylated histone and non-histone functions (Figure 1E and Supplementary Figure S1).

### RASEs in HDACi resistant and sensitive groups before and after treatment with HDACi

**FIGURE 2:**
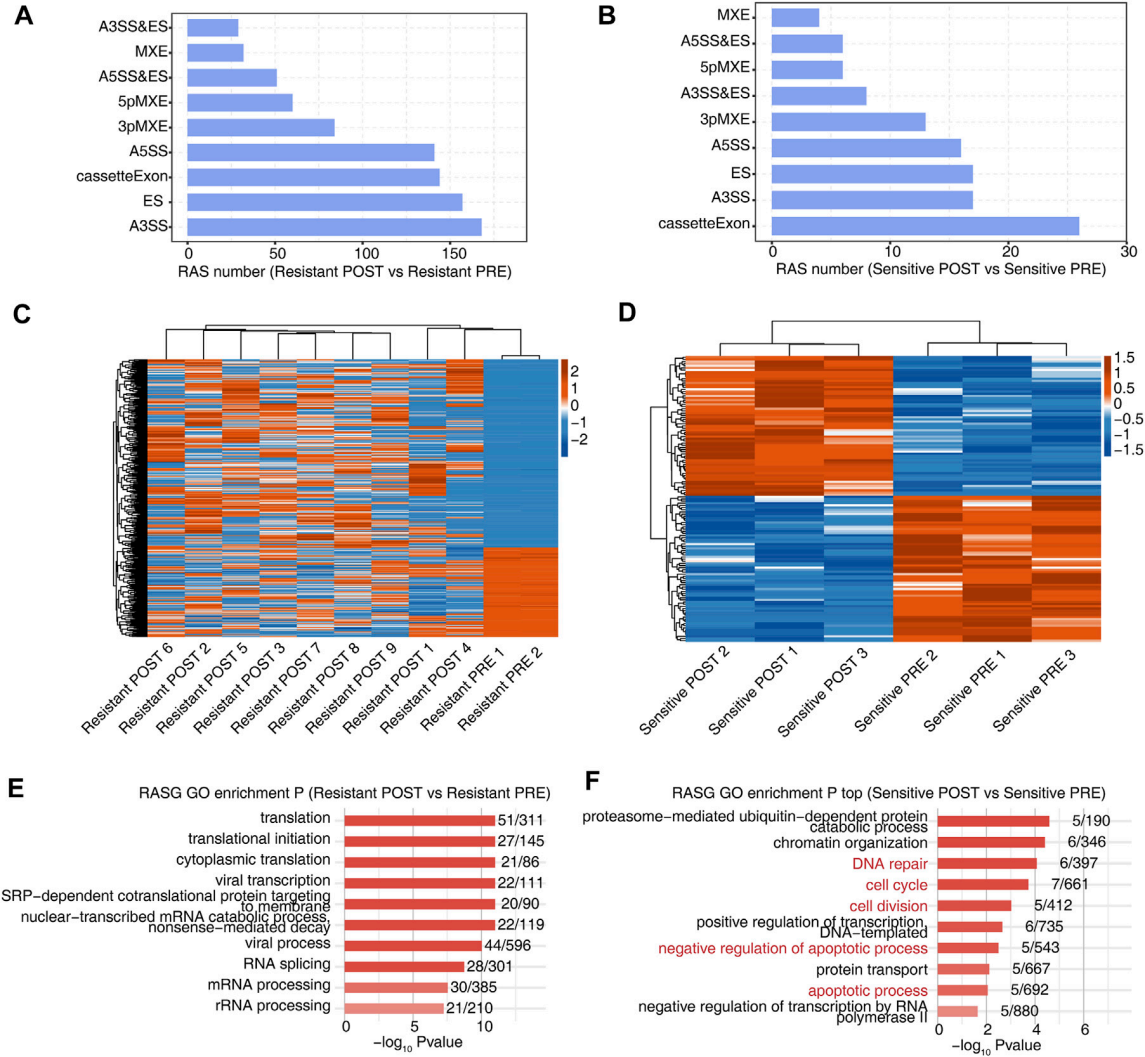
Alternative splicing before and after HDACi treatment were more heterogeneous in patients who were resistant than in those who were sensitive. **(A)** The bar plot showing the number of all significant regulated alternative splicing events between Resistant POST and Resistant PRE samples. *X*-axis: NIR RASE number. *Y*-axis: the different types of AS events. **(B)** The bar plot showing the number of all significant regulated alternative splicing events between Sensitive POST and Sensitive PRE samples. *X*-axis: NIR RASE number. *Y*-axis: the different types of AS events. **(C)** Hierarchical clustering heatmap of all significant NIR RAS based on splicing ratio of Resistant POST and Resistant PRE samples. **(D)** Hierarchical clustering heatmap of all significant NIR RAS based on splicing ratio of Sensitive POST and Sensitive PRE samples. **(E)** Bar plot showing the most enriched GO biological process results of the NIR RAS between Resistant POST and Resistant PRE samples. **(F)** Bar plot showing the most enriched GO biological process results of the NIR RAS between Sensitive POST and Sensitive PRE samples.

To further investigate the effects of HDACi treatment on AS, we identified AS events that were significantly different before and after treatment in sensitive and resistant groups (Figures 2A,B). Although more AS events were detected in the resistant group, this result may be related to the number of samples. Additionally, the heat map of the splicing ratio for AS events in the two groups showed that the resistant group exhibited stronger heterogeneity (Figures 2C,D). We analyzed the enrichment of GO and KEGG functional pathways for genes showing differential AS events before and after treatment. Notably, genes in which the AS events were significantly different before and after treatment in the resistant group were mainly enriched in pathways related to translation regulation (Figure 2E and Supplementary Figure S2A), whereas those in which the AS events were significantly different before and after treatment in the sensitive group were mainly enriched in DNA repair, apoptosis, chromatin organization, and other related pathways related to the effects of HDACi (Figure 2F and Supplementary Figure S2B).

### Significant RASEs and RBP/RASE network

**FIGURE 3:**
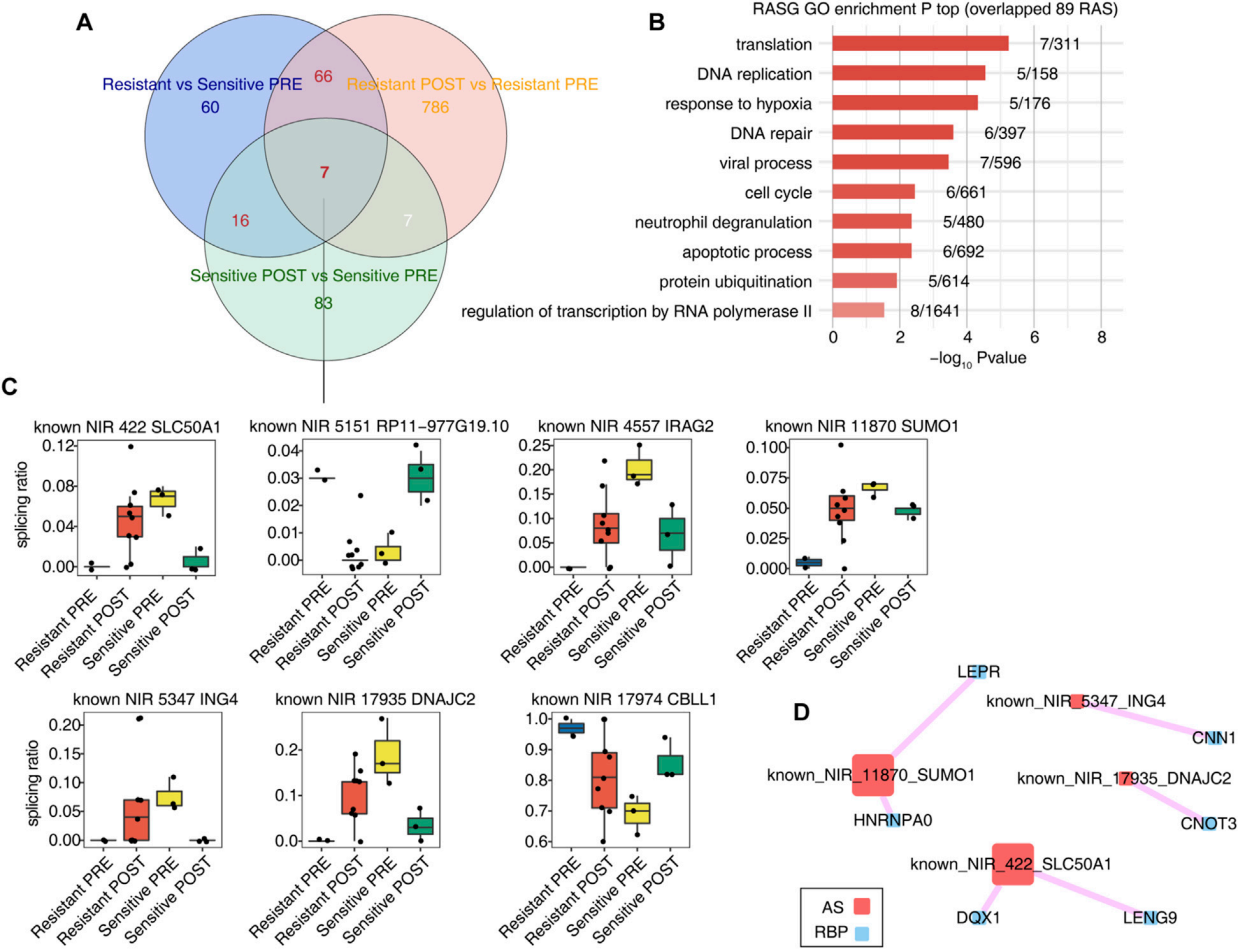
Several AS events associated with HDACi resistant were regulated completely opposite in the resistant group and the sensitive group before and after HDACi treatment. **(A)** Venn diagram showing the overlap of RAS between Resistant PRE and Sensitive PRE samples, RAS between Resistant POST and Resistant PRE samples, and RAS between Sensitive POST and Sensitive PRE samples. **(B)** Bar plot showing the most enriched GO biological process results of genes of 89 RAS with red lable in **(A) (C)** The co-disturbed network between expression of RBPs and splicing ratio of RASEs involved in Figure 3C was constructed. Pearson’s correlation| ≥0.7 and pvalue≤0.01 were retained. Circles represent RBP genes. Squares indicate RAS.

Differential splicing events in the sensitive and resistant groups before and after treatment were analyzed to identify those related to HDACi resistance (Figure 3A). We observed seven splicing events and significant differences between the sensitive and resistant groups before treatment. The functional pathways of the genes involved in these splicing events were mainly enriched in DNA repair, hypoxia response, apoptosis, and other pathways (Figure 3B). Figure 3C shows the seven splicing events associated with HDACi resistance in response to HDACi treatment in both the resistant and sensitive groups; these AS events were NIR_5151_RP11-977G19.10, NIR_4557_IRAG2, NIR_11870_SUMO1, NIR_5347_ING4, NIR_17935_DNAJC2, NIR_17974_CBLL1, and NIR_422_SLC50A1. Surprisingly, after treatment, these seven splicing events showed opposite changes in the sensitive and resistant groups.

The splicing ratio values of these seven AS events were significantly different before and after treatment in the resistant and sensitive groups, although the direction of this change was opposite. For example, in the known NIR_422 event, the splicing ratio increased in the resistant group and decreased in the sensitive group after treatment. A network correlation diagram of overall RBP/RASEs is shown in Supplementary Figure S3. Pearson correlation analysis was performed using the expression level of RBP and the splicing ratio of the seven splicing events, requiring a |correlation coefficient| greater than or equal to 0.7, and a *p* value less than or equal to 0.01. Notably, four of the seven splicing events had RBP/RAS covariation relationships (Figure 3D), implying potential regulatory functions of RBPs on RASEs events in response to HDACi.

## Discussion

AS is the process of reconnecting RNA exons produced by the transcription of mRNA precursors for genes via RNA splicing in various ways (Stamm et al., 2005). Abnormal splicing of genes is often observed in oncogenes and tumor-suppressor genes, and promotes tumor occurrence and development (Kozlovski et al., 2017; Sahin et al., 2021). HDACi can re-express inhibited regulatory genes in cancer cells and reverse their malignant phenotypes. Furthermore, HDACi can inhibit tumors by regulating biological activities, such as cell cycle arrest, angiogenesis, immune responses, aging, and apoptosis. Treatment with HDACi has become more effective in recent years (Singh et al., 2018; Chen et al., 2021), and has been shown to overcome tyrosine kinase inhibitor resistance induced by B-cell chronic lymphocytic leukemia-lymphoma-like 11 gene (*BIM*) deletion polymorphism in leukemia. The drug corrects the *BIM* precursor mRNA splicing in chronic myelogenous leukemia cell lines with *BIM* deletion polymorphisms by reducing the ratio of exon three- and exon 4-containing *BIM* transcripts, resulting in increased apoptosis (Rauzan et al., 2017; Siegfried and Karni, 2018). Use of HDACi is a promising therapy for CTCLs (Lopez et al., 2018). The HDACi included romidepsin and vorinostat in this study, of which 14 HDACi samples were treated with romidepsin and three HDACi samples were treated with vorinostat (Andrews et al., 2019). Romidepsin is a kinds of Class I HDACi, it can simultaneous inhibition of HDAC1/2. Vorinostat is a broad-spectrum HDACi. The two drugs may have a common mechanism of action in the treatment of CTCLs, both of which can partially reduce STAT3-dependent transcription, down-regulate the overexpression of interleukin (IL)-10, decrease the expression of IL-2 and IL-4, and increase interferon γ RNA expression (Tiffon et al., 2011; Xia et al., 2017).

This study is the first global analysis of AS in CTCL samples before and after HDACi treatment; 149 AS events with significant differences between HDACi-sensitive and -resistant groups before treatment were identified. Genes involved in these splicing events were enriched in DNA damage repair, apoptosis, and hypoxia response, which are related to histone and non-histone functions involving HDACi-dependent acetylation. Furthermore, we evaluated the different AS events between the HDACi-sensitive and -resistant groups before and after treatment. Major differences were observed between the sensitive and resistant groups. The AS event genes in the sensitive group were mainly enriched in DNA repair, apoptosis, chromatin tissue, and other pathways related to the functions of HDACi. Seven splicing events were also identified. The splicing ratios of these seven variable splicing events were significantly different between the resistant and sensitive groups before and after treatment. However, the direction of the change was opposite. This result suggests that the splicing of these genes yielded different response patterns to HDACi treatment; such splicing may be a key marker of HDACi sensitivity. This suggests that HDACi treatment regulates AS.

RBPs play an important role in the occurrence and development of tumors (Qin et al., 2020; Zhang and Li, 2021). Muys et al. found that p53-induced RBPs inhibited the splicing of carcinogenic CD44 variants in colorectal cancer (Muys et al., 2021). In this study, a co-change network of RBPs and AS events were established in CTCLs for the first time, and we analyzed the regulatory mechanisms through which RBPs affect HDACi sensitivity-related AS events. We found that *LEPR* and *HNRNPAO* had significant regulatory effects on NIR_11870_SUMO1, *CNN1* may regulate NIR_5347_ING4, *CNOT3* may regulate NIR_17935_DNAJC2, and *DQX1* and *LENG9* may regulate NIR_422_SLC5A1.

It has been confirmed that RBPs can not only inhibit tumor metastasis through protein-protein interactions and promote the splicing activity of splicing regulators, but also directly bind RNA and alter the splicing outcome (Hu et al., 2020). The potential regulatory relationships between RBPs and HDACi-sensitive RASEs were analyzed in this study. *LEPR* and *HNRNPAO* significantly affected NIR_11870_SUMO1, suggesting a potential regulatory relationship. *LEPR* gene polymorphisms are closely related to the risk of non-Hodgkin’s lymphoma (Uddin et al., 2010; Meininger et al., 2016). A *HNRNPA0* mutation can affect the expression pattern of phosphatidylinositol-3 kinase and extracellular signal-regulated kinase/mitogen-activated protein kinase signaling pathway, and increase the risk of cancer (Wei et al., 2015). Both these mechanisms may involve regulation of NIR_11870_SUMO1, which plays a pathogenic role in CTCLs. Additionally, *CNN1* may regulate NIR_5347_ING4, *CNOT3* may regulate NIR_17935_DNAJC2, and *DQX1* and *LENG9* may regulate NIR_422_SLC5A1. *CNN1* regulates DKK1/Wnt/β-catenin/c-Myc signaling pathway by activating the tissue inhibitors of metalloproteinases, and blocking the proliferation and metastasis of lung squamous carcinoma cells (Liu et al., 2021). Exome sequencing revealed mutations in *CNOT3* and the ribosomal genes *RPL5* and *RPL10* in acute lymphoblastic leukemia (De Keersmaecker et al., 2013; Bardelli et al., 2021). Furthermore, *DQX1* is reportedly associated with the prognosis of renal cell carcinoma (Qin et al., 2021). These RBPs may participate in the response of CTCLs to HDACi treatment through AS events, although the specific mechanisms still require evaluation.

This study has some limitations. First, the sample size is small. Moreover, this study is a proof of concept exercise, the selected HDACi resistance- or sensitivity-related AS events need to be further verified in a clinical settings. Second, the impact of RBPs on HDACi resistance or sensitivity and the specific mechanisms through which RBPs indirectly regulate these events should be further studied.

In summary, we identified seven indirect events of HDACi resistance or response in CTCLs in this study. We established a co-change network of RBP and AS events in CTCLs, and analyzed the potential regulatory effects of RBP on HDACi-related AS events. Our findings establish a theoretical foundation for the precise targeted treatment of CTCLs with HDACi.

## Data Availability

The datasets presented in this study can be found in online repositories. The names of the repository/repositories and accession number(s) can be found in the article/Supplementary Material.
