# Supplementary figures and images for: Genome-wide identification of alternative splicing associated with histone deacetylase inhibitor in cutaneous T-cell lymphomas
Source: Front Genet. 2022 Sep 6;13:937623. doi: 10.3389/fgene.2022.937623 (PMC9485882; doi:10.3389/fgene.2022.937623)

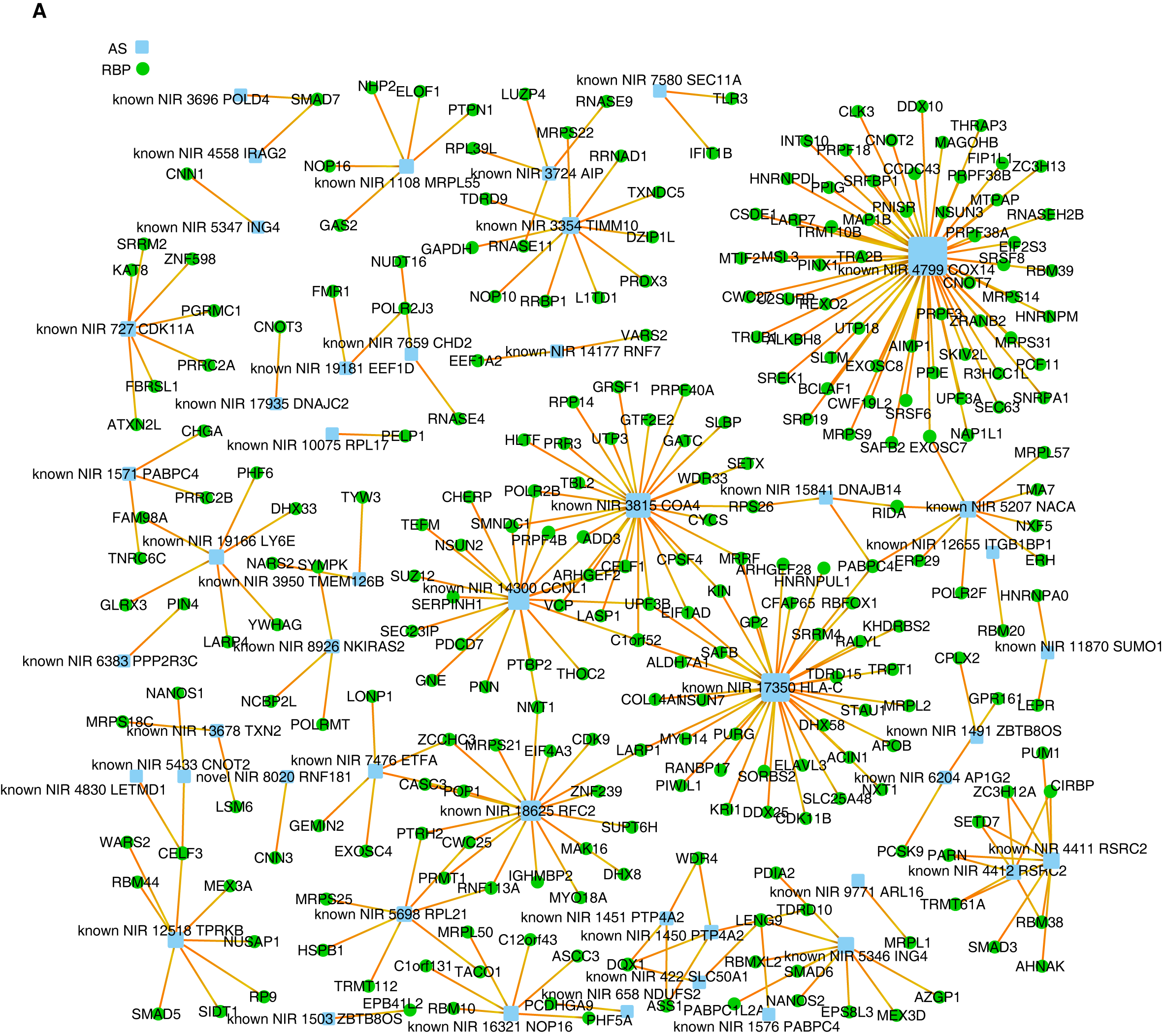

Supplement: Supplementary file 1 [file Image3.TIF]

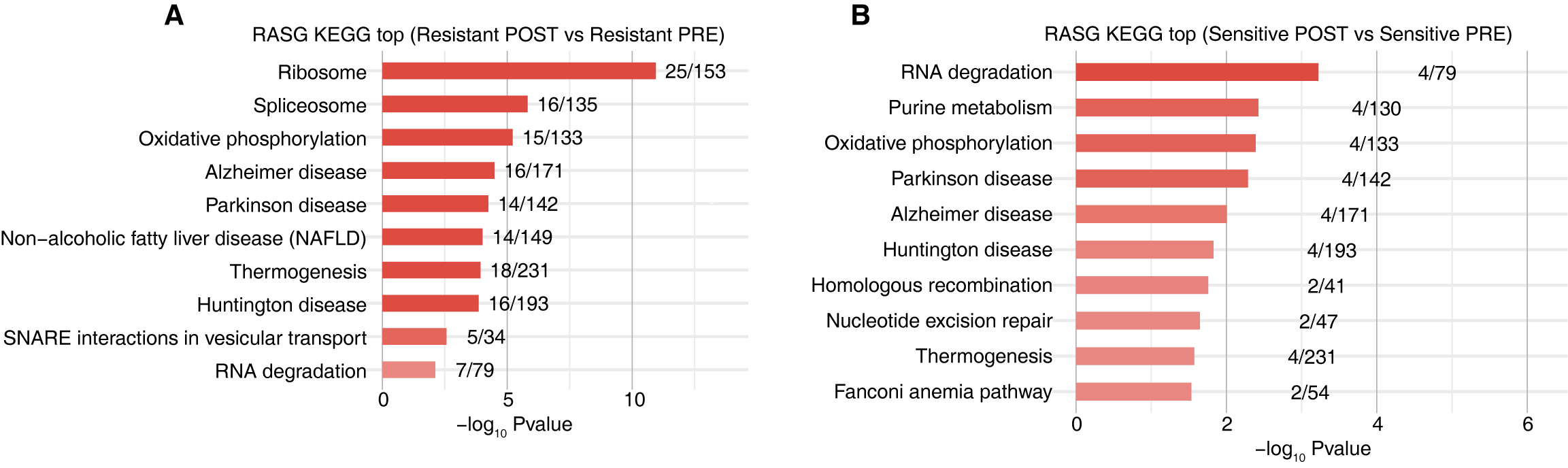

Supplement: Supplementary file 2 [file Image2.TIF]

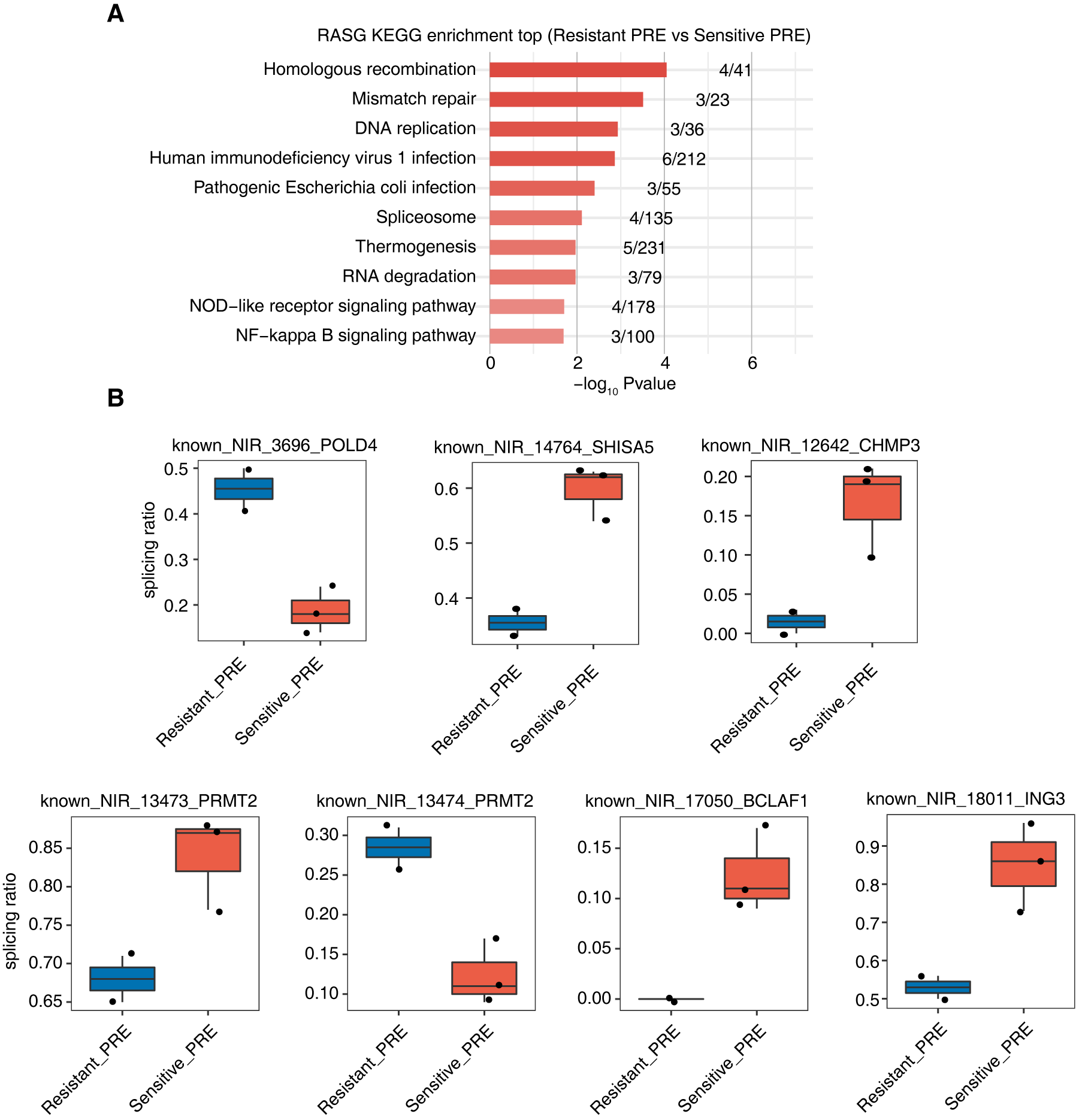

Supplement: Supplementary file 3 [file Image1.TIF]
